# *Ochratoxin A* Detection on Antibody- Immobilized on BSA-Functionalized Gold Electrodes

**DOI:** 10.1371/journal.pone.0160021

**Published:** 2016-07-28

**Authors:** Mihaela Badea, Laura Floroian, Patrizia Restani, Simona Codruta Aurora Cobzac, Marius Moga

**Affiliations:** 1 Fundamental, Prophylactic and Clinical Specialties Department, Transilvania University of Brasov, Brasov, Romania; 2 Automation and Information Technology Department, Transilvania University of Brasov, Romania; 3 Dipartimento di Scienze Farmacologiche e Biomolecolari, Università degli Studi di Milano, Milano, Italy; 4 Faculty of Chemistry and Chemical Engineering, Babes-Bolyai University, Cluj-Napoca, Romania; 5 Department of Medical And Surgical Specialties, Transilvania University of Brasov, Brasov, Romania; CSIR-Indian Institute of Toxicology Research, INDIA

## Abstract

Ochratoxin A (OTA)—a toxin produced by *Aspergillus carbonarius*, *Aspergillus ochraceus*, and *Penicillium verrucosum*—is one of the most-abundant food-contaminating mycotoxins. To avoid the risk of OTA consumption for humans and animals, the rapid detection and quantitation of OTA level in different commodities are of great importance. In this work, an impedimetric immunosensor for ochratoxin A (OTA) detection, a common toxic botanical contaminant, was developed via the immobilization of anti-OTA antibody on bovine serum albumin modified gold electrodes. A four-step reaction protocol was tested to modify the gold electrode and obtain the sensing substrate. All the steps of the immunosensor elaboration and also the immunochemical reaction between surface-bound antibody and ochratoxin A were analyzed using cyclic voltammetry and electrochemical impedance spectroscopy. Modification of the impedance due to the specific antigen-antibody reaction at immunosensor surface, was used in order to detect ochratoxin A. Linear proportionality of the charge transfer resistance to the concentration of OTA allows ochratoxin A detection in the range of 2.5–100 ng/mL.

## Introduction

Ochratoxin A (OTA) is a mycotoxin produced by *Aspergillus ochraceus*, *Aspergilius niger* and *Penicillium verrucosum*, found as contaminants of a variety of food, such as cereals, coffee beans, beans, grapes and dried fruit. OTA is one of the most toxic and widespread compound from the ochratoxins group [1,2].

Studies have shown that OTA can have specific toxicological effects such as nephrotoxic, teratogenic, neurotoxic, hepatotoxic and immunotoxic, and it is believed to cause increased oxidative stress at a cellular level [3,4]. The concerns about OTA contamination determined different research groups to develop high-performance detection techniques for quality assessment.

Fourty years ago, OTA was identified as a corn contaminant in USA, being produced by *Penicillium viridicatum* wrestling. Ever since, OTA has been found through the whole world: in regions with cold and temperate climate it is produced by *Penicillium verrucosum* and by *Aspergillus carbonarius* and also in regions with hot and tropical climate, where it is produced by *Aspergillus ochraceus*. *Penicillium verrucosum* is the specific fungi of stocked cereals, while *Aspergillus ochraceus* is the most common champignon of the green coffee, spices, cocoa, soya, peanuts, rice and *Aspergillus carbonarius* is contaminant of grapes [5]. Even though crop fungi contamination can take place pre and post-harvest, OTA synthesis is believed to be performed during the storage period. The mechanism of the OTA biosynthesis by different fungi and the coding genes are not well known. It is clear that OTA production depends on the toxicogenic power of the strains but also on the common practices during the food processing. For example, prevention of OTA production in the cereals is achieved by controlling the humidity conditions during the filling of the grain elevator and during storage, knowing that a water activity higher than 0.8 (aw) is favorable to the development of *Penicillium verrucosum*. OTA contamination of grapes in the wine yards is explained only by the fruit damages made by insects or by the harvest devices, because, by default, *Aspergillus* strains are not pathogenic for the wine yard itself.

Analytical methods for OTA quantification follow the same steps as the ones for the quantification of mycotoxins: sampling and sample preparation, extraction, purification (clean-up), separation and detection. European Commission regulation No. 401/2006 from 23 February 2006 lays down the methods of sampling and analysis used for the official control of the amount of mycotoxins in foodstuffs. The separation methods are coupled with the detection technique that is sensitive enough to fulfill the legally imposed limits, but they require sample extraction and clean-up and they are rather expensive and demand specially trained personnel. Specific clean-up methods includes immunoaffinity columns [6,7]. After this step, HPLC was recommended in order to detect the occurence of ochratoxin in food commodities: coffee, pepper, chili, prickly ash, cinnamon, aniseed, fennel, curry powder and cumin [7–9].

Chemical and enzymatic assays were used with success in small-molecule detection [10], but nowadays the immunoassays are considered novel screening methods which provide sensitive detection and can be used by non-specialists under field conditions. Although there is a great emphasis on their selectivity, the main drawback is still their cross-reactivity. Scientific literature indicated that false-negative results are rarely reported, but false-positive results are more frequent and depend on several factors like temperature, pH, sample viscosity or ionic strength [11]. Without sample clean-up or extraction before the testing, matrix effects might be expected leading to significant overestimation of mycotoxin concentration, especially in colorimetric detection when color samples are tested. Therefore, positive results should be confirmed with the conventional analytical methods to avoid misinterpretations.

Electrochemical sensors and biosensors are an alternative solution due to their design and method of detection. For example, OTA was detected using square wave voltammetry at a glassy carbon electrode (GeE) [12]. Limit of detection of this assay was of 0.02 μg/kg and the sensor was used for the detection of OTA extracted from wine sample using antibody modified magnetic nanoparticles.

A biosensor for the detection of OTA was designed via the immobilization of HRP on screen printed carbon electrode (SPCE) using a polypyrrole matrix [13].

Immunosensors have also been developed for effective and fast screening of OTA in foodstuffs. These are based on a variety of detection techniques such as electrochemical [14,15], optical (e.g surface plasmon resonance [16], optical waveguide light-mode spectroscopy technique [17], fluorescence [18,19] etc) and acoustic methods (quartz crystal microbalance immunosensors [20]).

Kinetics and mechanisms of electron-transfer processes that correspond to the biocatalytic reaction occurring at modified electrodes and also interfacial properties changes of modified electrodes [21,22], such as those linked to biorecognition events involving antibody–antigen binding, at modified surfaces [23] can be analyzed with the powerful tool of electrochemical impedance spectroscopy (EIS).

Electrochemical detection systems seem most promising thanks to their high sensitivity, feasibility of low cost, low endogenous background, compatibility with portability and miniaturization.

Several reviews have been published on the use of EIS in biosensors [24,25]. Using EIS method, there were monitored the changes in the electrical properties at the (bio)sensors interface.These changes can be associated with specific binding events due to the recognition between an analyte and a ligand. Antibodies and more recently, aptamers [26,27], have been used as biorecognition elements in biosensors with EIS detection. Literature data indicated EIS methods for ochratoxin detection from different matrices (Table 1).

**Table 1:** Sensors used for ochratoxin A detection.

Type of biosensor	Methods used to characterize the electrodes	Linear range	Detection limit (and other parameters)	Refe-rences
Highly sensitive and reusable electrochemical impedimetric aptasensor	CV, EIS	1.25 ng/L—500 ng/L	0.25 ng/L	[28]
Direct competitive immunosensor	the substrate the p-benzoquinone generated enzymatically was detected by differential-pulse voltammetry		in wines was of 0.11 ± 0.01 ng/L	[29]
A Langmuir-Blodgett (polyaniline (PANI)-stearic acid (SA)) film based highly sensitive and robust impedimetric aptasensor	SEM, FTIR, CV, EIS, contact angle measurements	0.1 ng/mL -10 ng/mL, and 1 μg/mL-25 μg/mL	0.1 ng/ml in 15 min can be reused ∼13 times	[30]
A self-assembled monolayer (SAM) of 11-amino-1-undecanethiol (AUT) has been fabricated onto a gold (Au) substrate to co-immobilize anti-ochratoxin-A antibodies (AO-IgGs) and bovine serum albumin (BSA)	SEM, CV, DPV, EIS	over 0.5–6.0 ng/dL	0.08 ng/dL using 3σb/m criteria, response time of 30 s, regression coefficient of 0.999	[31]
Nanostructured zinc oxide (Nano-ZnO) film has been deposited onto indium-tin-oxide (ITO) glass plate for co-immobilization of rabbit-immunoglobulin antibodies (r-IgGs) and bovine serum albumin (BSA)	XRD, FTIR, SEM, EIS	0.006–0.01nM/dm^3^	0.006 nM/dm^3^, response time as 25s, regression coefficient of 0.997	[32]

In this work, an impedimetric immunosensor for the detection of ochratoxin A was developed via the immobilization of the anti-OTA antibody gold electrodes previously modified with a cross-linked film of bovine serum albumin. A four-step reaction protocol was tested in order to modify the gold electrode and obtain the sensing substrate. All the steps of the immunosensor elaboration and also immunochemical reaction between surface-bound antibody and ochratoxin A were analyzed using cyclic voltammetry and electrochemical impedance spectroscopy. Modification of the impedance appeared at immunosensor surface due to the specific antigen-antibody reaction was used in order to detect ochratoxin A. Specifically, the increase of the electron-transfer resistance (R_ct_) at the interface was correlated with OTA concentration in the range of interest.

## Materials and Methods

### Materials and reagents

Gold printed electrode DRP-250AT was purchased from DS Dropsens (Spain). The electrodes (SPCEs) incorporate a conventional three-electrode configuration, which comprises a disk-shaped Au working electrode (1.6 mm diameter, 0.0196 cm^2^ geometrical area), Au counter electrode and silver pseudo-reference electrode.

N-Hydroxysuccinimide (NHS—PubChem CID: 80170), N-(3-dimethylaminopropyl)-N’-ethylcarbodiimide (EDC—PubChem CID: 15908), potassium ferrocyanide (K_4_Fe(CN)_6_—PubChem CID: 71309461), potassium ferricyanide (K_3_Fe(CN)_6_—PubChem CID: 26250), Ochratoxin A (PubChem CID: 442530) were purchased from Sigma–Aldrich, St. Louis (USA). Bovine serum albumin (BSA) Crystalized 100% was purchased from Mann Research Laboratories Division of Becton Dickinson & Co NY (USA) and monoclonal antibody anti-Ochratoxin A from Novus Biologicals (Canada). Acetic acid (PubChem CID: 176), sodium acetate trihydrate (PubChem CID: 23665404), acetonitrile (PubChem CID: 6342), sulphuric acid (PubChem CID: 1118) and ethanolamine (PubChem CID: 700) were purchased from Chemical Company, Iasi (Romania).

### Buffers and solutions

Acetate buffer pH 5.6, comprising of 0.1 M acetic acid and 0.1 M sodium acetate was prepared using distilled deionized water. BSA 5 mg/mL in acetate buffer and antibody solution 5 μg/mL in acetate buffer were prepared.

A solution containing 0.1 M KCl, 5 mM K_3_[Fe(CN)_6_] and 5 mM K_4_[Fe(CN)_6_] was used in cyclic voltammetry and electrochemical impedance spectroscopy measurements. Blocking buffer solution consisted of ethanolamine 0.1 M in water and NHS and EDC solution were also prepared in deionized water.

OTA 5 mg/mL was diluted in different concentration in acetate buffer.

### Apparatus

An Autolab PGSTAT100 Eco Chemie (Netherlands) potentiostat was used to carry out the impedance spectra at 10 mV sinusoidal ac potential perturbation in the frequency range from 10^4^ to 10^−1^ Hz, superimposed on +0.178 V dc potential, that is the potential of the ferrocyanide/ferricyanide couple [Fe(CN)6]^4−^/^3−^. The spectra were taken in 1mM ferrocyanide/ferricyanide solution (1:1 mixture) in 0.1M KCl as background electrolyte at room temperature. All the measurements were performed in a solution of The FRA 4.9 software calculates and records the real and imaginary parts of electrochemical impedance (Z’ and Z”) together with the phase and represents them in Nyquist and Bode diagrams.

EIS using the classic ferricyanide/ferrocyanide redox probe was frequently used for quantitation of various molecules with biosensors, including with real samples. A few recent examples for ochratoxin A were included in Table 1. The ionic strength of the solution was always the same and controlled by the composition of the electrolyte. All measurements were done in 5 mM potassium ferri/ferrocyanide in 0.1M KCl, before and after incubation with the standard or sample solution, as indicated in Experimental-Solutions and Buffers section.

Based upon the principles of electrochemical spectroscopy, the equivalent electric circuit that best fits the experimental data was found and optimum electrical parameters were obtained: electrical resistance of the solution, charge transfer resistance, constant phase element and Warburg impedance. For each modified electrode, the impedance spectra were recorded before and after incubation with the standard or sample solution. The variation in the R_ct_ following incubation with standard/sample was calculated as ΔR_ct_ = R_ct_(after)-R_ct_(before) and was correlated with the concentration of aflatoxin in the sample.

### Electrode modification and immobilization of the antibodies

Before modification, Dropsens gold electrode have been subjected to electrochemical pretreatment by applying 10 potential cycles between -0.3 and +1.5 V / pseudo silver reference electrode with 100 mV/s scan rate in 0.5 M H_2_SO_4_ solution until the voltammogram characteristic for a clean Au surface was obtained.

The clean gold electrode was first modified using BSA/EDC/NHS, creating a cross-linked film that prevents the non-specific binding of OTA on gold, and allows further covalent attachment of antibody. Electrode modification was performed as described by Polonschii et al. [33], 5 mg/ mL BSA (50 μL), 20 μL of 0.4M EDC and 20 μL of 0.1 M NHS were mixed and allowed to stand 5 minutes at room temperature. Afterwards, 10 μL of this mixture were evenly spread on the working electrode and allowed to stand at room temperature for 30 minutes in a humid atmosphere. The electrode was rinsed with a lot of water and dried in air.

Next, the terminal carboxylic groups on BSA film were activated by dropping 10 μL of a 1:1 mixture of EDC/NHS onto the sensor surface, allowing the reaction to proceed, allowing it to react for 40 min at room temperature in a humid dark room. The electrode surface was rinsed after each step thoroughly with copious amounts of water for removing the unbound material. After this, the antibody anti-OTA immobilization was done by covering the modified electrode surface with a 10 μL droplet of the 5 μg/mL antibody solution in acetate buffer, allowing it to react in a water-saturated atmosphere for 1 h at room temperature. After incubation the electrode was rinsed in water to remove unbound antibodies and 75 μL ethanolamine 1M solution was drop cast onto the modified surface and incubated 15 min with the aim to deactivate the remaining succinimide groups and also to block unreacted active sites. These modified electrodes can then be stored dry several days at 4°C without a decrease in the sensitivity, or they can be subjected to immunochemical reaction.

**Fig 1:**
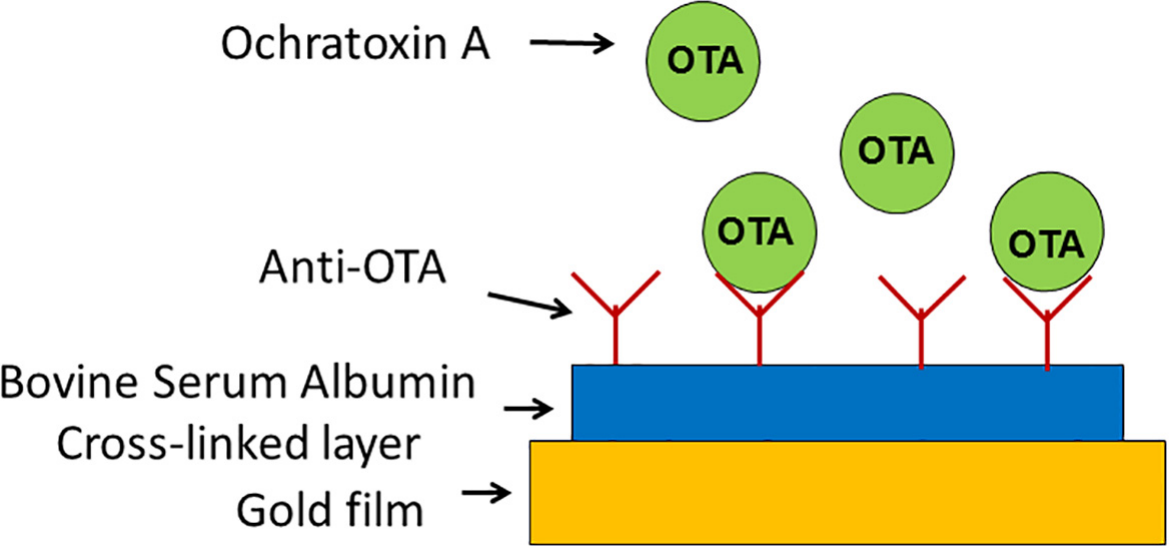
Schematic outline of the electrochemical immunosensor for OTA determination.

A schematic representation of the analytical principle of this electrochemical immunoassay is shown in Fig 1.

For the OTA measurement, 10 μL of either sample or OTA standard solutions of different concentrations in acetate buffer pH 5.6 were pipetted onto the working electrode area and allowed to stand at room temperature for 45 min in a humid atmosphere (to prevent evaporation). The immunosensor was rinsed with a large amount of water before the electrochemical measurements.

Parameters such as the incubation time and the amount of antibody/electrode were optimized to obtain good analytical characteristics, appropriate for Ochratoxin A detection in real samples.

## Results and Discussion

### Electrochemical measurements

We have performed Faradic electrochemical impedance spectroscopy measurements using the classic redox probe ferricyanide/ferrocyanide, at the formal potential of this reversible redox couple, in order to induce the slightest possible perturbation in the system, as was recommended by Bard and Faulkner [34].

**Fig 2:**
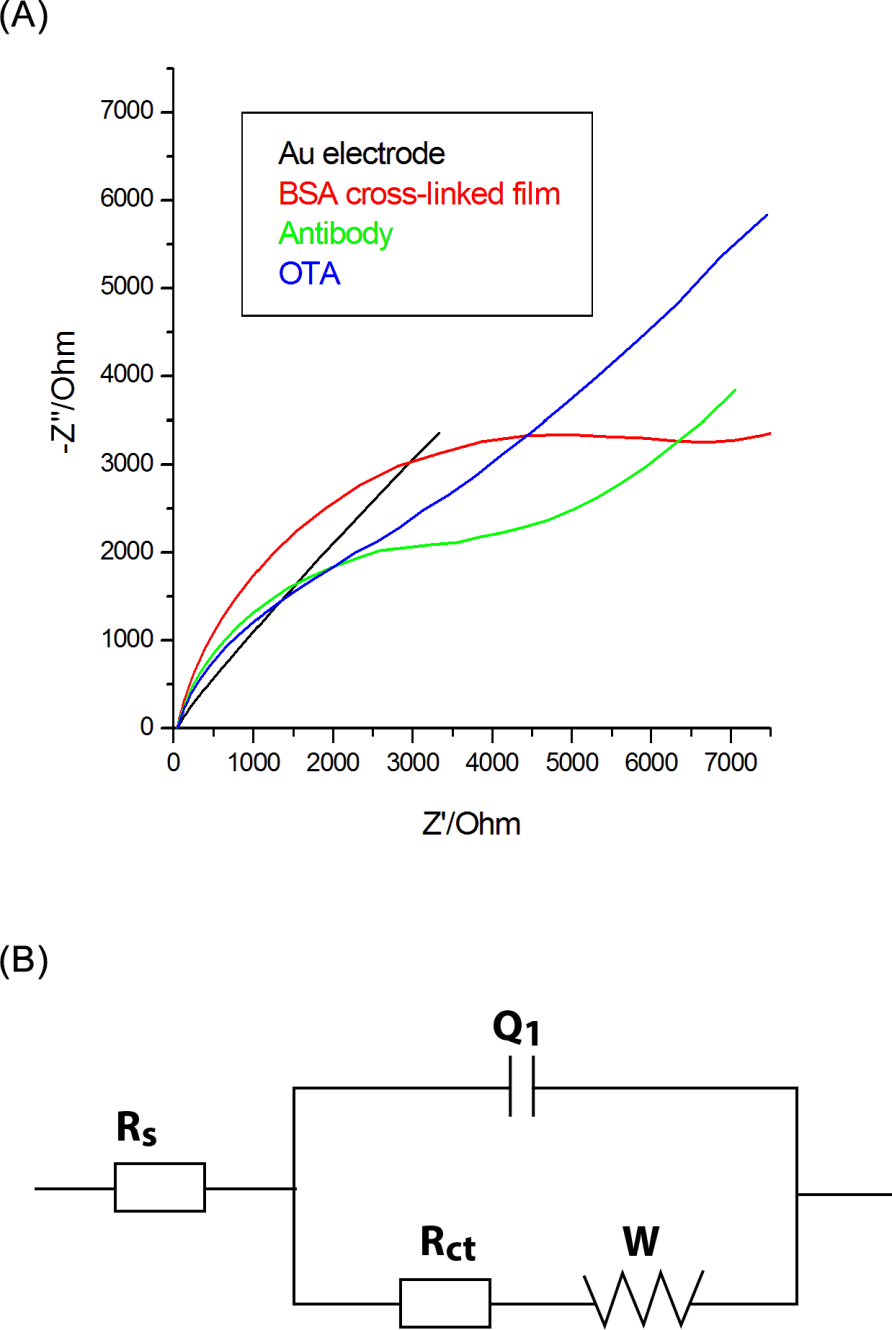
A) EIS Nyquist spectra of each modification electrode step, at 10 mV sinusoidal ac potential perturbation, 10^4^ to 10^−1^ Hz frequency, ferricyanide/ferrocyanide redox couple and B) Equivalent electric circuit.

Fig 2A displays the Nyquist impedance spectra recorded upon the stepwise process of electrode modification, providing specific information on the barrier properties and the changes at the interface sensor-solution throughout the biosensor building process. The bare gold electrode showed an extremely small semicircle domain (black curves), implying a very low electron-transfer resistance of the redox probe.

After the grafting of the BSA film on the electrode, R_ct_ increased significantly (red curves), due to the deposition on the electrode surface of an organic layer with negatively charged terminal carboxylic groups COO^−^ (the isoelectric point of BSA being 4.7).

The protein layer acts as a physical and an electrostatic barrier for the [Fe(CN)_6_]^4−/3−^ anions, preventing redox probe to reach the electrode surface and slowing down the electron transfer kinetic between the probe and the electrode. Similar approaches were also used by Radi and colab. [26]. Next, antibodies were covalently immobilized onto the BSA modified electrode and a remarkable drop in the R_ct_ was observed (blue curves) because the negative charge of BSA- modified electrode is partly neutralized by the covalent attachment of the antibody. Afterwards, the R_ct_ increased when the sensor was used for OTA detection (magenta curves), as the OTA binding to surface-bound antibody created an additional barrier to the electron transfer at the interface.

Variants of equivalent electrical circuits were tested before choosing the most appropriate circuit for our experimental curves from Fig 2A. For this circuit, by using the facilities of FRA 4.9 software, we analysed each individual response for each experimental step and we considered the data where we obtained the low Chi-square values. The impedance data were fitted to equivalent circuit shown in the Fig 2B that includes the solution resistance (R_s_), the charge transfer resistance (R_ct_), the constant phase element (Q) and the Warburg impedance element (W).

Ideally, W and Rs represent the properties of the electrolyte and diffusion features of the redox probe in this solution and they are not affected by modifications at the electrode surface. Q value depends very much on the dielectric properties of the layer that separates the electrode surface and the ionic charges, the thickness of the separation layer and surface area of the electrode. A large increase in the Q value was noted when bare Au electrode was covered with BSA-EDC-NHS layer, whereas a Q decrease was observed upon further attachment of the antibody or of OTA to the sensor interface.

R_ct_ value depends on the insulating properties at the electrolyte/electrode interface. R_ct_ changes were much larger than those in other impedance components, and thus R_ct_ can be considered an adequate signal for the determination of the interfacial properties for the prepared immunosensor.

The experiments were run in triplicated and statistical analysis was used. Table 2 shows the average values of equivalent circuit parameters for all the steps of the immunosensor elaboration and also for the immunochemical reaction between ochratoxin A and its antibody.

**Table 2:** Averages values of the equivalent circuit parameters for various steps of the immunosensor.

Modification	Rs (Ω cm^2^)	Q (10^−6^μF)	n	Rct (Ω cm^2^)	W (10^−6^ Ω cm^2^)
**Bare electrode**	4.10	142	0.92	1.267	806
**BSA-cross linked film**	4.52	513	0.85	413	635
**Anti-OTA Antibody**	4.29	238	0.88	157	683
**OTA 10 ng/mL**	3.97	122	0.86	307	724

EIS is a sensitive tool for monitoring affinity interactions at surfaces, but particularly due to this high sensitivity it is highly recommended to confront the impedance results with other electrochemical techniques (cyclic voltammetry, linear swept voltammetry or differential pulse voltammetry), and to record a good parallel control of the samples [35].

**Fig 3:**
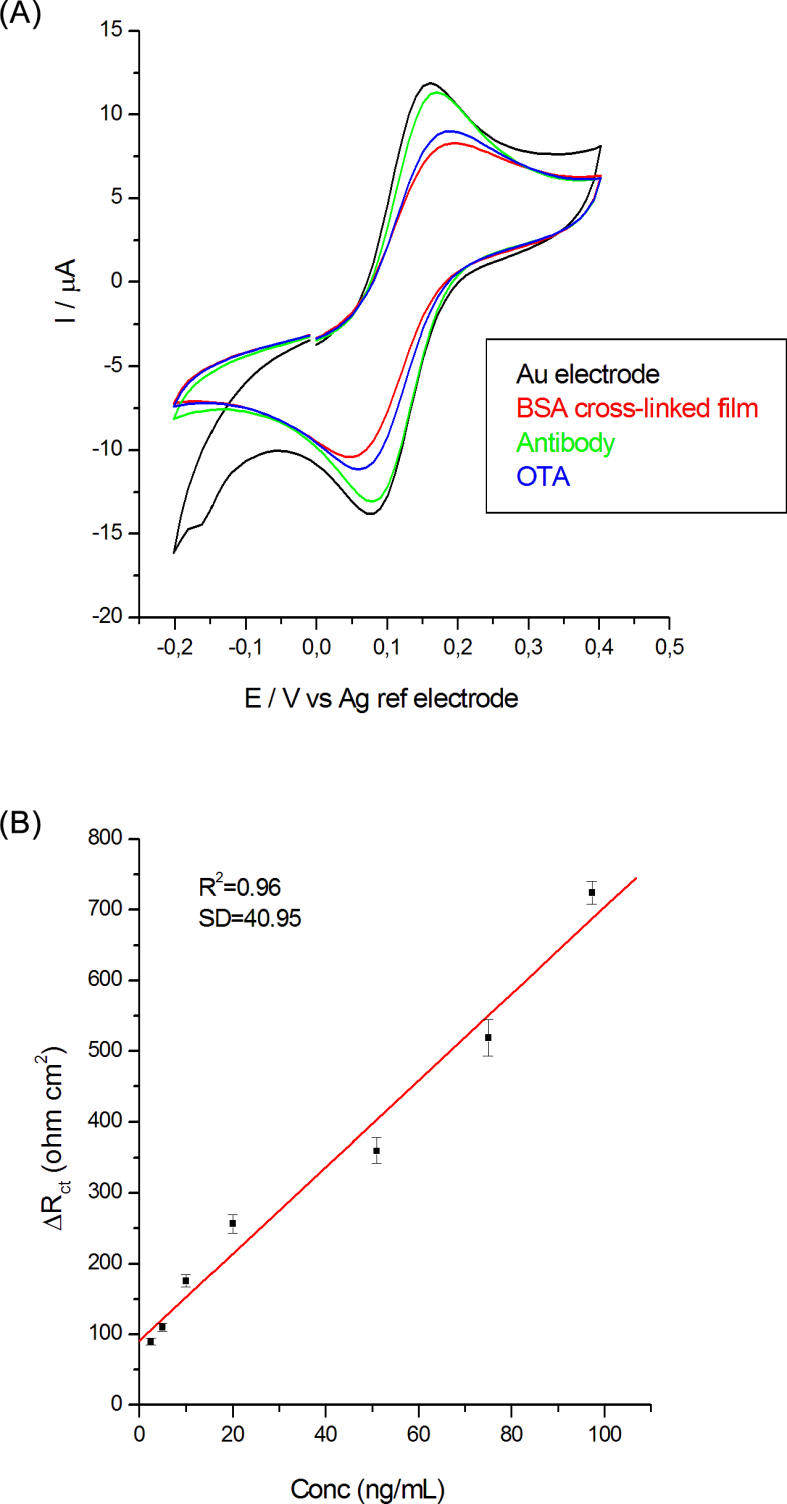
A) CVs of the sample at 0.1 V/s scan rate in ferricyanide/ferrocyanide redox couple, after each step of the immunosensor build-up and B) OTA calibration curve.

It was observed that our results are consistent with the cyclic voltammetry curves shown in Fig 3A.

The cyclic voltammograms of soluble electroactive species provide a convenient tool for monitor the various stages of the immunosensor building on gold electrode. The CV-s were performed after all the step of electrode modification and also after toxin adding on electrode surface. Fig 3A shows the CV-s in solution of 5 mM ferricyanide in 0.1 M KCl, 100 mV/s scan rate, for initial gold electrode, BSA/EDC/NHS modified electrode, antibody anti-OTA/BSA/EDC/NHS modified electrode and after formation of immunochemical complex on the surface of the anti-OTA/BSA/EDC/NHS modified electrode after 15 ng/mL OTA solution addition.

The cyclic voltammograms are strongly affected by the deposited layers, the difference between the anodic and cathodic peak potentials does not remained constant, whereas the peak current modifies significantly. It can be seen initial the characteristic quasireversible redox cycle for a bare Au electrode and after its functionalization with BSA/EDC/NHS, the electron transfer between the redox probe and electrode surface was severely affected and an obvious decreasing of the anodic and cathodic peaks was observed. After the Ab immobilization on the functionalized electrode surface, the peak currents of the redox couple of ferricyanide/ferrocyanide increases again. Immunochemical reaction of OTA molecules with the antibody film determined a decrease in the Faradaic response. It was observed also an increase in the peak-to-peak separation between the cathodic and anodic waves of the redox probe, indicating that the electron-transfer kinetics of ferricyanide/ferrocyanide is obstructed. All the observations are in accordance with results of EIS analyses about studied electrodes and these two techniques allow a good parallel control of the samples [35].

To evaluate the immunochemical reaction between antibody anti-OTA and OTA, we exposed the anti-OTA/BSA-EDC-NHS/Au electrode to various OTA concentrations. It was found an increase for R_ct_ parameter with the adding of OTA (Table 3).

**Table 3:** Average values of the equivalent circuit parameters for various OTA concentrations.

Conc. OTA (ng/mL)	Rs (Ω cm^2^)	Q (10^−6^μF)	n	Rct (Ω cm^2^)	W (10^−6^ Ω cm^2^)	ΔRct (Ω cm^2^)
100	4.16	172	0.85	1451	712	724
75	4.22	128	0.86	884	744	519
50	3.98	120	0.90	759	866	370
20	4.06	120	0.90	431	804	256
10	3.97	122	0.86	307	724	176
5	4.27	137	0.83	641	820	110
2.5	4.01	92	0.84	934	887	90

**Fig 4:**
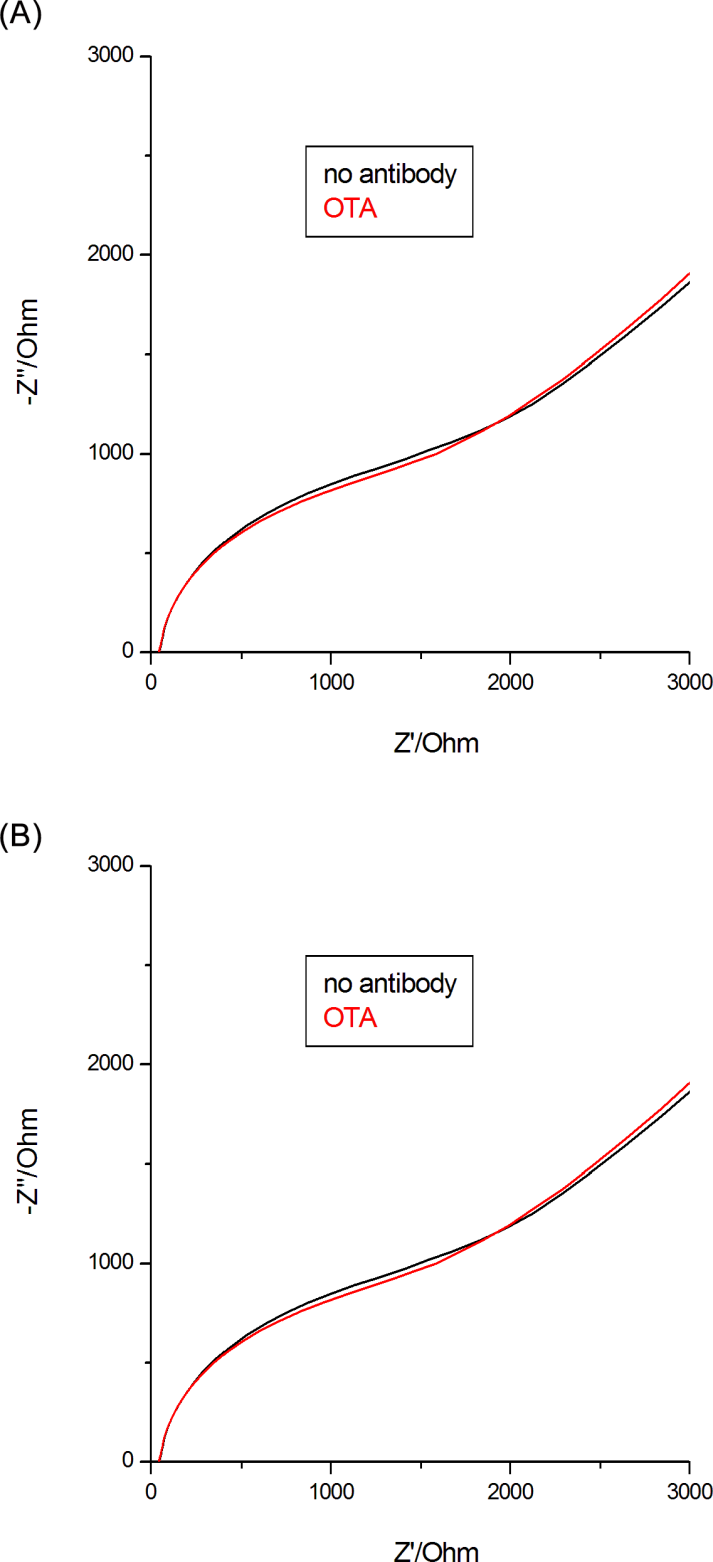
Nyquist plots at 10 mV sinusoidal ac potential perturbation, ferricyanide/ferrocyanide redox couple, for response of sensor with an antibody specific for OTA (A) or of sensor without antibody (B).

The difference between R_ct_ values before and after incubation with OTA is considered the analytical signal ΔR_ct_ = R_ct_(after)-R_ct_ (before). As it can be seen in Fig 3B, there is a steady linear increase in ΔR_ct_ with the OTA concentration. The calibration curve in Fig 4 was further used for determining the OTA concentration in plant extracts samples.

### Immunosensor specificity

In this work, we immobilized monoclonal antibody anti-Ochratoxin A from Novus Biologicals (Canada) in order to propose a rapid method for ochratoxin A detection using antibody-immobilized on BSA-functionalized gold electrodes. We chose to do as this because, even that the cost of biosensor could increase, this choosing minimized the cross-reactivity (previous tests were done to check cross reactivity using aflatoxin B1—data not shown in this paper–and no cross-reactivity was observed).

Some control experiments were performed with a sensor fabricated without antibody, aiming to confirm that the impedance changes were indeed due to specific interaction between OTA and its antibody, and were not caused by nonspecific adsorption. This second sensor was prepared using an identical protocol with identical conditions, buffers, concentrations, etc. as used for the specific antibody electrodes.

Fig 4A shows the impedance spectra recorded for a sensor before and after the incubation of 10 ng/mL OTA and Fig 4B for supplemental control experiment that uses antibody-free device. In this last case, no obvious impedance changes were detected upon the incubation with OTA, which confirm that the observed impedance changes are due to specific antibody-OTA interactions.

We have proven the specificity of the interaction with OTA and the fact that non-specific adsorption effects are insignificant by making EIS determinations with a biosensor unmodified with antibody anti-OTA.

### OTA detection in plant extracts samples

The liquorice obtained from local market was crushed into mortar with pestle and 1 g of powder was mixed for 6 minutes with 5 mL of acetate buffer (pH 5.6). The mixture was kept at rest for 5 minutes and then was filtered through absorbent filter paper and 0.2 μm Nylon syringe filter. This extract (stock solution) was further diluted 1:1000 in acetate buffer in order to be used for the experiment.

50 μL plant extract was mixed with 50 μL OTA in different concentrations before analysis with the electrochemical immunosensor. A volume of 75 μL of each sample was dropped on the surface of antibody- modified electrodes and allowed to incubate for 30 minutes. The Nyquist diagrams were recorded before and after incubation of the sensors with the plant extracts. The impedance data were fitted to equivalent circuit shown in Fig 2B, and the solution resistance, the electron transfer resistance, the constant phase element and the Warburg impedance element were determined (Table 4).

**Table 4:** Values of the equivalent circuit parameters for electrodes with plant extracts.

Sample	Rs (Ω cm^2^)	Q (10^−6^μF)	n	Rct (Ω cm^2^)	W (10^−6^ Ω cm^2^)	ΔRct (Ω cm^2^)
Sample 1 OTA 5 ng/mL	4.01	107	0.87	261	670	122
Sample 2 OTA 10 ng/mL	4.02	153	0.87	409	848	150.5

Using OTA calibration curve and ΔR_ct_ (the difference between R_ct_ values before and after incubation with plant extract) the OTA concentration of plant extracts was determined (Table 5).

**Table 5:** OTA concentration in spiked plant extracts.

Sample	ΔRct (Ω cm^2^)	Concentration OTA (ng/mL)	Recovery degree of OTA (%)
Sample 1	122	4.63	92.6%
Sample 2	150.5	9.82	98.2%

In this way, our new label-free, sensitive, cost-effective and fast EIS immunosensor can be utilized for OTA detection. The sensor based on screen-printed gold electrodes was easily modified with a cross-linked film of BSA that further serves as “anchor” for the covalent immobilization of the anti-OTA antibody. The casting of the protective BSA layer on the gold electrode prevents any nonspecific binding between OTA and the gold surface.

The specific interaction between antibody and OTA induces an increase in electron transfer resistance at the interface sensor-solution that is correlated with the concentration of OTA in the sample. The detection of OTA was achieved by EIS on the linear range 2.5–100 ng/mL. Obtained results have the advantage of larger linear range, which include the maximum levels of OTA allowed by EC in various food products and are similar with some others obtained using immunosensors and EIS/SPR detection [36]. The he immunosensor can be further optimized and our next work will consider amplification strategies of the analytical signal in order to improve the sensitivity of this method for OTA detection. One possible future option to optimize the detection of these mycotoxins is the use of aptamers, where Catanante and colab. [37] obtained very promising results, with better dynamic range. Another possibility is to employ PEC (label-free photoelectrochemical) platform recommended as strategy for fabrication of label-free biosensor by Yang and colab. [38].

## Conclusion

A new label-free immunosensor for ochratoxin A detection was developed. This sensitive, fast and cost-effective sensor based on a screen gold electrode, which was easily modified in order to immobilize the monoclonal antibody anti-OTA, induced an increase in electron transfer resistance at the interface immunosensor-solution that is related to ochratoxin A concentration in the sample. The method could be successfully used for detection of ochratoxin A from plant extracts using bioanalysis and biosensing.
